# Supplementary material for: Transgene Was Silenced in Hybrids between Transgenic Herbicide-Resistant Crops and Their Wild Relatives Utilizing Alien Chromosomes
Source: Plants (Basel). 2022 Nov 22;11(23):3187. doi: 10.3390/plants11233187 (PMC9741405; doi:10.3390/plants11233187)
Supplement: Supplementary file 1 [file plants-11-03187-s001.zip › Sup Fig.pdf]

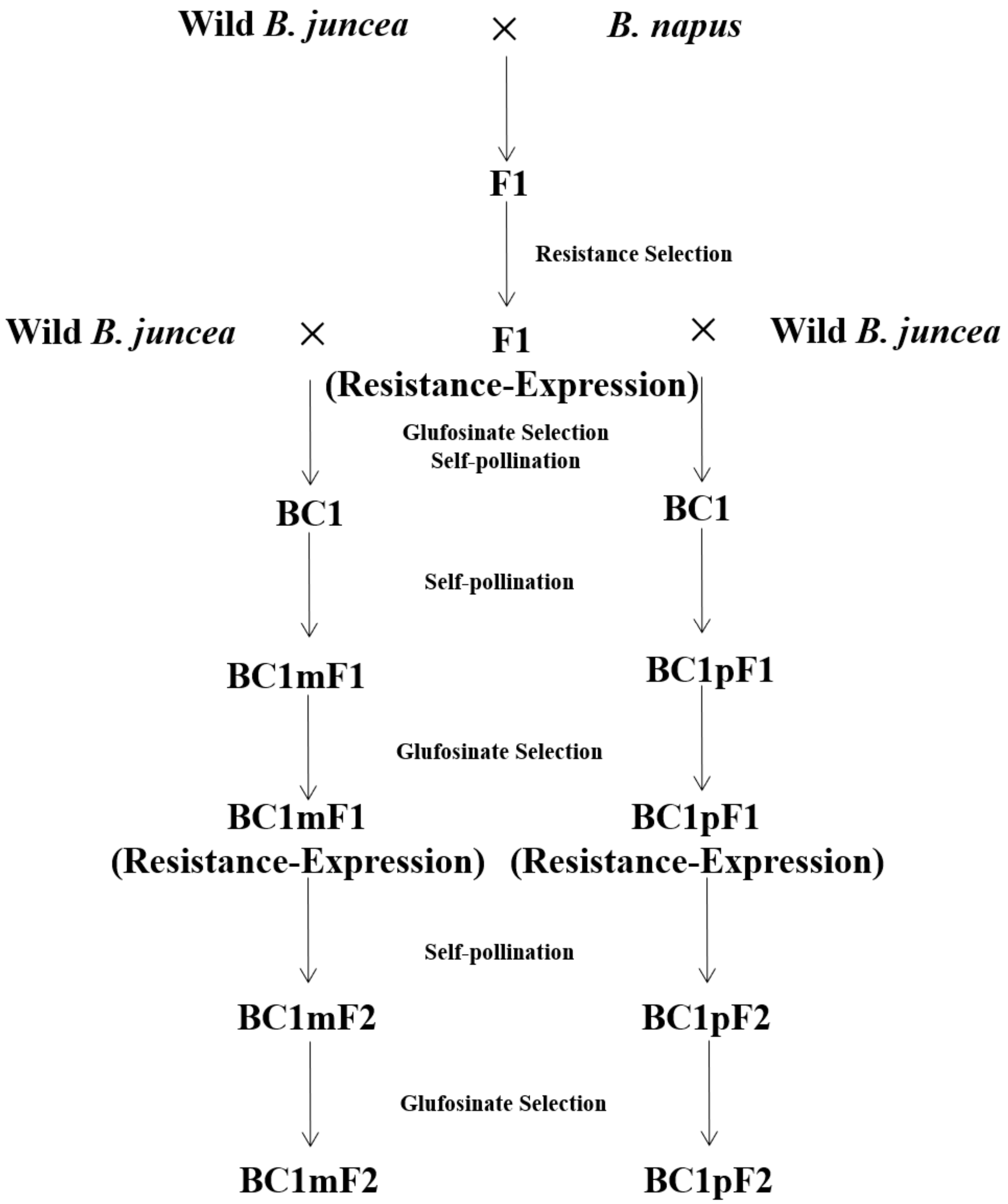

Figure S1 Crossing scheme of hybridization and backcrossing between wild *Brassica juncea* and transgenic glufosinate-resistant *Brassica napus* and self-pollination of the first backcross generation (BC1)

Note: Combinations involved in this study are indicated as maternal plants× paternal plants. Plants in front of × are always maternal plants, and populations in the back of × are always paternal plants. m denotes backcross progeny obtained with wild *Brassica juncea* as maternal plants. p denotes backcross progeny obtained with wild *Brassica juncea* as paternal plants. *BC* = backcross generation.
