# Supplementary material for: Transgene Was Silenced in Hybrids between Transgenic Herbicide-Resistant Crops and Their Wild Relatives Utilizing Alien Chromosomes
Source: Plants (Basel). 2022 Nov 22;11(23):3187. doi: 10.3390/plants11233187 (PMC9741405; doi:10.3390/plants11233187)
Supplement: Supplementary file 1 [file plants-11-03187-s001.zip › Sup Methods.pdf]

Supplementary Methods

1. Transmission and expression of transgenes in backcross progenies

1.1 Glufosinate screening

For the two different backcross combinations (BC1mF and BC1pF), the ten mother plants in each generation that displayed the highest seed fertility for each lineage were chosen. At least 50 filled seeds from each of the ten mother plants were selected randomly and each seed sown directly into individual plastic pots (6-cm diameter) containing growth media (mixture of garden soil and peat at 1:1 (v/v) ratio) for the duration of the investigation. Plants were grown as described previously by Song et al [14].

1.2 Extraction of DNA template

Extract the total DNA of the material by SDS method:

① Weighed 0.2 g of the leaves as the test material, froze and grinded them into powder with liquid nitrogen, and transferred them to about 0.3 ml of a 2.0 ml centrifuge tube;

② Added 600 μL of 65 °C preheated extraction solution, mix well, and then bathed at 65 °C for 30 minutes, during which shook for 3-4 times until the sample solution is dark green;

③ Added 150 mL NaAc (1/4 of the extract) and shook well;

④ Added 600 mL of chloroform: isoamyl alcohol (24:1), shook well, centrifuged at 12000 r/min for 5 min, and transferred 500 μL of supernatant to another 2.0ml centrifuge tube

⑤ Added anhydrous ethanol 1000 μL (twice the volume of supernatant), evenly mixed, 12000 r/min, centrifuged for 5 min;

⑥ Discarded the supernatant, added 400 mL volume fraction of 70% ethanol, mixed it slightly (or place it for about 10min), 12000 r/min, centrifuged for 2-3 minutes, poured the alcohol and dry it at room temperature;

⑦ Added 200-400 μL TE or double distilled water (1μL RNA enzyme can be added)

into the centrifuge tube containing DNA, and gently flicked it with hand to dissolve the sediment- Store at 20 °C.

See Table S2 for PCR system

Took 3 μL PCR products were detected by agarose gel electrophoresis.

2. Insert site of *PAT* and its promoter

2.1 Correctness verification of designed primers

The whole genome DNA of glufosinate resistant transgenic *Brassica napus* was used as a template. The procedure is as follows:

① See Table S4 for PCR reaction system;

② The PCR reaction conditions of promoter (CaMV35s) and its flanking sequence were as follows:

94℃	5min	
94℃	30s	35Cycles
62℃	30s	
72℃	1min	
72℃	10min	

③ Took 3 μL PCR products were detected by agarose gel electrophoresis.

3. qPCR analysis

3.1 RNA extraction

When the tested plants grew to 4-6 leaf stage, 10 resistant-gene-expressing and resistant-gene-silencing plants of different backcross progenies were sprayed with glufosinate. Samples were taken at 0h, 6h and 12h after spraying, and then frozen in liquid nitrogen and stored in - 80 °C refrigerator.

RNA extraction was carried out according to the instructions of the RNA Extraction Kit Biospin plant total RNA extraction kit of bioflux. The specific operating steps are as follows:

1 Reagent preparation: added in lysis before operation β- Mercaptoethanol to a final concentration of 5%. Take 500 μL lysis, added to a 1.5ml centrifuge tube, and 50 μL plantaid, standby.

2 Sample pretreatment: after grinding an appropriate amount of fine powder of plant tissue in liquid nitrogen, weighed an appropriate amount of fine powder and put it into the above 1.5ml centrifuge tube equipped with lysis and plantaid, immediately shook it violently until there is no obvious granule, and left it at room temperature for 5min.

3 The lysates were centrifuged at 1300rpm for 10min, and the fragments that could not be lysed and bound with polysaccharide polyphenols were precipitated, and 420 μL lysate supernatant was transferred to a new 1.5ml centrifuge tube.

4 Accurately estimated the supernatant volume of the lysate, add 0.5 times the volume of absolute ethanol, blew and mixed immediately, and did not centrifuge.

5 The mixture was sucked into the spin column, the spin column was sleeved with a centrifuge tube, and centrifuged at 1300rpm for 1min

6 Discarded the liquid in the outer sleeve and add 600 to the spin column μL PG buffer, stood at room temperature for 30s, centrifuged at 12000rpm for 30s, and discarded the liquid in the liquid receiving pipe.

7 Added 600 to spin column μL wash buffer, centrifuged at 12000rpm for 30s, and discarded the liquid in the liquid receiving pipe. Joined 600 μL wash buffer to wash again.

8 Then the empty column was centrifuged at 12000 rpm for 1min, and the wash buffer was removed as much as possible to prevent the residual ethanol in the wash buffer from inhibiting the downstream reaction.

9 Moved the spin column into a new 1.5ml centrifuge tube, and add the regeneration buffer 50 in the center of the membrane μ l. Total RNA was obtained by standing at room temperature for 1min.

Since RNA was extremely unstable and easy to decompose, the extracted RNA is stored in a - 80 °C refrigerator.

3.2 cDNA synthesis

According to the reverse transcriptase Primescript ™ RT reagent kit with gDNA eraser

(perfect real time) (Takara company) instructions to prepare reaction solution:

1. Prepared the reaction mixture on ice according to Table S5. Prepare the mixture and pack it into the reaction tube, and finally add RNA sample.

2. The above reaction solution was treated at 42 ℃ for 2 min.

3. The reaction solution should be prepared according to Table S6. The mixed solution should be prepared according to the amount of reaction number + 2, and then each tube should be divided into 10 μL. After mixing, the reverse transcription reaction should be carried out immediately.

4. The above reaction solution was treated at 37 ℃ for 15min and 85 ℃ for 5S.

5. Stored the cDNA at -20 ℃ for standby.

6. qPCR primers and amplification reaction system of *PAT* genes was set according to Table S7&8.

4. Methylation site detection

4.1 DNA bisulfite treatment

DNA methylation was performed by bisulfite treatment according to the instructions of methyldetectortm bisulfite modification kit. The amount of DNA was 500 pg^{-2} μg. The optimum concentration is 200-500ng. The experimental process is as follows:

1 Buffer preparation

① Conversion Buffer

Took 1 tube of CT conversion reagent and added:

ddH$_2$O 900 μL

M-Dilution Buffer 300 μL

M-Denaturation Reagent 175 μL

Shook it on the shaking table for 10 minutes at room temperature. It should be used immediately after preparation, or it can be stored at - 20 ℃ for one week.

② Preparation of m-wash-buffer

Add 24 ml of 100% ethanol to the m-wash buffer to configure a usable wash buffer.

2 Conversion reaction

① Prepare CT conversion buffer solution.

② In the order of 200 μL Add the following reagents to the PCR tube:

CT Conversion Buffer 130 μL

DNA 20 μL

If the volume of the DNA sample was less than 20 ml, used water to make up for the difference and mix the sample.

③ Placed the sample in the PCR instrument, and the procedure is as follows:

98℃ 10min

64℃ 2.5h

Stored the product at 4 ° C (up to 20 h).

3 Upper column desulfurization and DNA purification

①Add 600μL m-binding buffer to the zymo spin IC column and placed the column in the collection tube.

② Add the obtained PCR product to the zymo spin IC column with m-binding buffer and mix the samples.

③ Centrifugal (≥ 1000 × g) 30s, pour the waste liquid.

④ Added all the reaction liquid into the collection tube, centrifuged at 10000 rpm for 30s.

⑤ Transferred 200 μ L DNA wash buffer into the tube, 10000 rpm, and centrifuged for 30s.

⑥ Added 200 μL m-wash buffer to the column, full speed (≥ 1000 × g) Centrifuge for 30 seconds and discard the waste liquid.

⑦ Added 200 μL m-desnlphonation buffer, room temperature (20-30℃) for 15-20min, full speed (≥ 1000 × g) Centrifuge for 30s and discarded the waste liquid.

⑧ Joined 200 μ L m-wash buffer, full speed (≥ 1000 × g) Centrifuged for 30 seconds and added 200 μ L m-wash buffer, full speed (≥ 1000 × g) Centrifuged for 30 seconds.

⑨ Added 10μL of m-elution buffer to the matrix of the column, put the column into a 1.5ml centrifuge tube, and centrifuged (≥ 1000 × g) 60s, and DNA was recovered. DNA was stored at - 20 ℃, and the amount of each PCR was 2-4 μL eluent.

4.2 PCR amplification

① Template: DNA extracted after methylation treatment of Roundup resistant transgenic *Brassica napus* and wild *Brassica juncea* forward and reverse BC1;

② According to the promoter (CaMV35s) and *PAT* gene sequence, methylation amplification was carried out in three stages. The designed primers were shown in Table S9;

③ See Table S10 for PCR reaction system;

④ The methylation PCR reaction conditions of promoter (CaMV35s) and *PAT* gene were as follows:

94°C 5min

94°C 30s

52.5°C 30s 50Cycles

72°C 2.5min

72°C 10min

PCR products were detected by agarose gel electrophoresis.

5. Fitness analysis

5.1 Planting methods

Uniformly-sized 100 plants of Resistant-gene-expressing and 100 plants of Resistant-gene-silencing in each self-pollinated generation (from 10 mother plants) were selected after glufosinate screening, and then transplanted individually into pots (25 cm diameter) containing the same growth media. Plants were grown in greenhouse conditions according to Song et.al [14].

5.2 Calculation methods of composite fitness

The fitness-associated traits of *Brasscia juncea* were defined as '1', and every other type was assigned a fitness associated traits based on its ratio to the value assigned to progenies of the transgenic *Brassica napus* and wild *Brassica juncea*. The composite fitness was the mean of eight relative fitness estimates from the vegetative to mature stages [14].
