# Supplementary material for: Transgene Was Silenced in Hybrids between Transgenic Herbicide-Resistant Crops and Their Wild Relatives Utilizing Alien Chromosomes
Source: Plants (Basel). 2022 Nov 22;11(23):3187. doi: 10.3390/plants11233187 (PMC9741405; doi:10.3390/plants11233187)
Supplement: Supplementary file 1 [file plants-11-03187-s001.zip › Sup Tab 11190948.pdf]

Table S1 DNA PCR Primers for *PAT* gene

Primer name	Primer sequence	Application
PAT-F	5'- AGGACAGAGCCACAAACACCAC-3'	PCR amplification
PAT-R	5'- ACCAACATCATGCCATCCACCA-3'	PCR amplification

Table S2 The system of polymerase chain reaction for *PAT* gene

Reaction components	Concentration	Volume (μL)
Template	1.0μg·μL^{-1}	1.0
Primer P1	10.0μM	1.0
Primer P2	10.0μM	1.0
BU-Taq MIX	2×	7.0
ddH$_2$O	-	10.0
Total Volume	-	20.0

Table S3 The specific primers designed according to the promoter (CaMV35S) of *PAT* gene and its flanking sequences

Primer name	Primer sequence	Application
CaMV`L-F	5'- CGAGGAGGTTTCCGGATATTAC-3'	PCR Amplification
CaMV`L-R	5'-GAACAGAGAGGAGGTGAAGAAG-3'	PCR Amplification

Table S4 The system of PCR with the promoter (CaMV35S) of *PAT* gene and its flanking sequences

Reaction components	Concentration	Volume (μL)
Template	1.0μg·μL^{-1}	1.0
Primer 1	10.0μM	1.0
Primer 2	10.0μM	1.0
Takara Ex Taq	5U·μL^{-1}	0.3
Ex Taq Buffer	10×	2.5
Mg2+	25mM	2.0
dNTP	2.5mM	2.0
ddH2O	-	15.2
Total Volume	-	25.0

Table S5 Preparation for the reverse transcription master mix of *PAT* genes in the backcross generation between wild *Brassica juncea* and transgenic glufosinate-resistant *Brassica napus*

Contents	Dosage of contents∕μL
5×gDNA Eraser Buffer	2.0
gDNA Eraser	1.0
Total RNA	7.0
Total Volume	10.0

Table S6 Reaction system of the reverse transcription master mix of *PAT* genes in the backcross generation between wild *Brasscia juncea* and transgenic glufosinate-resistant *Brassica napus*

Contents	Dosage of contents∕μL
Reaction solution after pretreatment in Table S5	10.0
PrimeScript RT Enzyme Mix I	1.0
RT Primer Mix	1.0
5×PrimeScript Buffer 2（for Real Time）	4.0
RNase Free dH_2O	4.0
Total Volume	20.0

Table S7 qPCR primers for *PAT* and *HMG* genes in the fourth progenies of the first backcross generations between glufosinate-resistant transgenic *Brassica napus* and wild *Brassica juncea*

Primer names	Primer sequence
PAT-F	5'- AGGACAGAGCCACAAACACCAC-3'
PAT-R	5'- ACCAACATCATGCCATCCACCA-3'
HMG-F	:5'-GGTCGTCCTCCTAAGGCGAAAG-3';
HMG-R	: 5'-CTTCTTCGGCGGTCGTCCAC-3';

Table S8 qPCR amplification reaction system of *PAT* genes in the backcross generation between wild *Brassica juncea* and transgenic glufosinate-resistant *Brassica napus*

Contents	Dosage of contents⁄µl
10×Ex Taq Buffer	2.5
$MgCl_2$ （25mM）	2
dNTP Mix （2.5mM）	2
Primer-F （10uM）	1
Primer-R （10uM）	1
Ex Taq （5U/µl）	0.2
cDNA	1
dH_2O	15.3
Total Volume	25

Table S9 The primers for DNA methylation PCR of transformant promoter (CaMV35S) and *PAT* gene

Primer name	Primer sequence	Length of amplification(bp)
MCa.P -F	5'- TTTTATGGAGTTAAAGATTTAAAT-3'	368
MCa.P -R	5'-AAAATCCATCTTTAAAACCACTATC -3'	
MCa.P1 -F	5'- GGAAAAGGAAGGTGGTTTTTATAA-3'	325
MCa.P1 -R	5'- AACCTAATCTCAACTAATCTCCTCTCC -3'	
MCa.P2 -F	5'- TTTTATTTGGAGAGGATAGGGTATT -3	237
MCa.P2 -R	5'- ACCTCAACAACCAACCAAAAATAT -3	
MCa.P3 -F	5'- TAGATATTTTTGGTTGGTTGTTGAG -3	351
MCa.P3 -R	5'- ACTAACAACTCAAAATCCCTTTACC -3	

Table S10 The system for PCR of transformant promoter (CaMV35S) and *PAT* gene

Reaction components	Concentration	Volume (µL)
DNA template	1.0µg·µL-1	2.0
P-F	15.0µM	1.0

P-R	15.0μM	1.0
Takara Taq Ex HS	5U·μL-1	0.4
Takara Taq E Buffer	10×	2.5
Mg^{2+}	25mM	2.0
dNTP	2.5mM	2.0
ddH2O	-	14.1
Total Volume	-	25.0

Table S11 Fitness-associated Traits measured at two life cycle stages

Life stage Vegetative	Reproductive
**Plant height:** Height from the base of the plant to the tip of the plant at maturity	**Seed weight:** The weight of seed/plant
**Stem diameter:** Main stem diameter of each plant measured at maturity	**Number of silique / plant:** number of siliques/plant at maturity
**number of effective branches:** Number of branches with silique which carried seeds	**Silique length:** silique length at lower part of plant measured at maturity stage (sample of at least 20 siliques per plant).
**Dry above-biomass:** measured at final harvest as described by Song et al., 2021 [14]	**Seed number/silique:** number of the filled seeds in silique at measuring silique length
